# Apocrine Hidrocystoma of the Lower Eyelid: A Case Report

**DOI:** 10.7759/cureus.114504

**Published:** 2026-08-13

**Authors:** Moe Wada, Isao Nagata, Ayumi Matsumoto, Toshihito Mitsui, Natsuko Kakudo

**Affiliations:** 1 Plastic and Reconstructive Surgery, Kansai Medical University, Hirakata, JPN

**Keywords:** apocrine hidrocystoma, benign tumor, eyelid, plastic surgery, surgical excision

## Abstract

Apocrine hidrocystoma is an uncommon benign cystic neoplasm of apocrine gland origin that most frequently occurs in the head and neck region, particularly around the eyelids. Because its clinical appearance is often nonspecific, it may be difficult to distinguish from other benign eyelid cystic lesions before excision. We report the case of a 33-year-old man who presented with a painless, skin-colored subcutaneous mass on the medial aspect of the left lower eyelid. The patient reported no seasonal change in lesion size, and no conjunctival abnormality was observed. The lesion was clinically suspected to be a benign cystic tumor and was excised under local anesthesia. Histopathological examination revealed a cyst lined by one to two layers of cuboidal to columnar epithelial cells with papillary infoldings and characteristic apical decapitation secretion. An outer myoepithelial cell layer was also identified. There was no cytological atypia or mitotic activity, and the lesion was diagnosed as an apocrine hidrocystoma. The postoperative course was uneventful, with no evidence of recurrence at the one-month follow-up. This case illustrates the clinicopathological features of apocrine hidrocystoma and emphasizes the role of histopathological examination in the definitive diagnosis of clinically nonspecific eyelid cystic lesions.

## Introduction

Apocrine hidrocystoma is an uncommon benign cystic neoplasm of apocrine gland origin. Mehregan described the lesion under the term apocrine cystadenoma in 1964 [1]. It occurs predominantly in the head and neck region, particularly around the eyelids where the glands of Moll are located, although rare cases have also been reported on the trunk, axillae, genital region, and extremities [2]. Clinically, the lesion usually presents as a solitary, slowly enlarging, asymptomatic cystic nodule and is often difficult to distinguish from other benign eyelid cystic lesions on clinical examination alone [3,4].

This diagnostic difficulty is clinically relevant because simple aspiration or needle puncture may be followed by recurrence [4]. In addition, pigmented variants may clinically resemble malignant eyelid tumors, including basal cell carcinoma, sebaceous carcinoma, and malignant melanoma [4]. Therefore, appropriate surgical excision and histopathological examination are important not only for establishing the diagnosis but also for avoiding inadequate treatment and excluding malignancy.

Histopathological examination is essential for definitive diagnosis because characteristic findings-including papillary infoldings, apical decapitation secretion, and an outer myoepithelial cell layer-cannot be identified clinically [3,5]. Although apocrine hidrocystoma has traditionally been regarded as a retention cyst, histopathological and immunohistochemical studies support an apocrine (Moll gland) origin for many eyelid hidrocystomas [5].

Herein, we present a case of lower-eyelid apocrine hidrocystoma as an educational example illustrating its clinical presentation, characteristic histopathological features, differential diagnosis, and surgical management.

## Case presentation

A 33-year-old man presented to the Department of Plastic and Reconstructive Surgery with a painless mass located at the medial aspect of the left lower eyelid, which he had first noticed three years earlier. He had no significant medical or family history. During the clinical course, the lesion had remained stable without episodes of erythema, infection, or changes in size associated with seasonal or ambient temperature variation.

**Figure 1:**
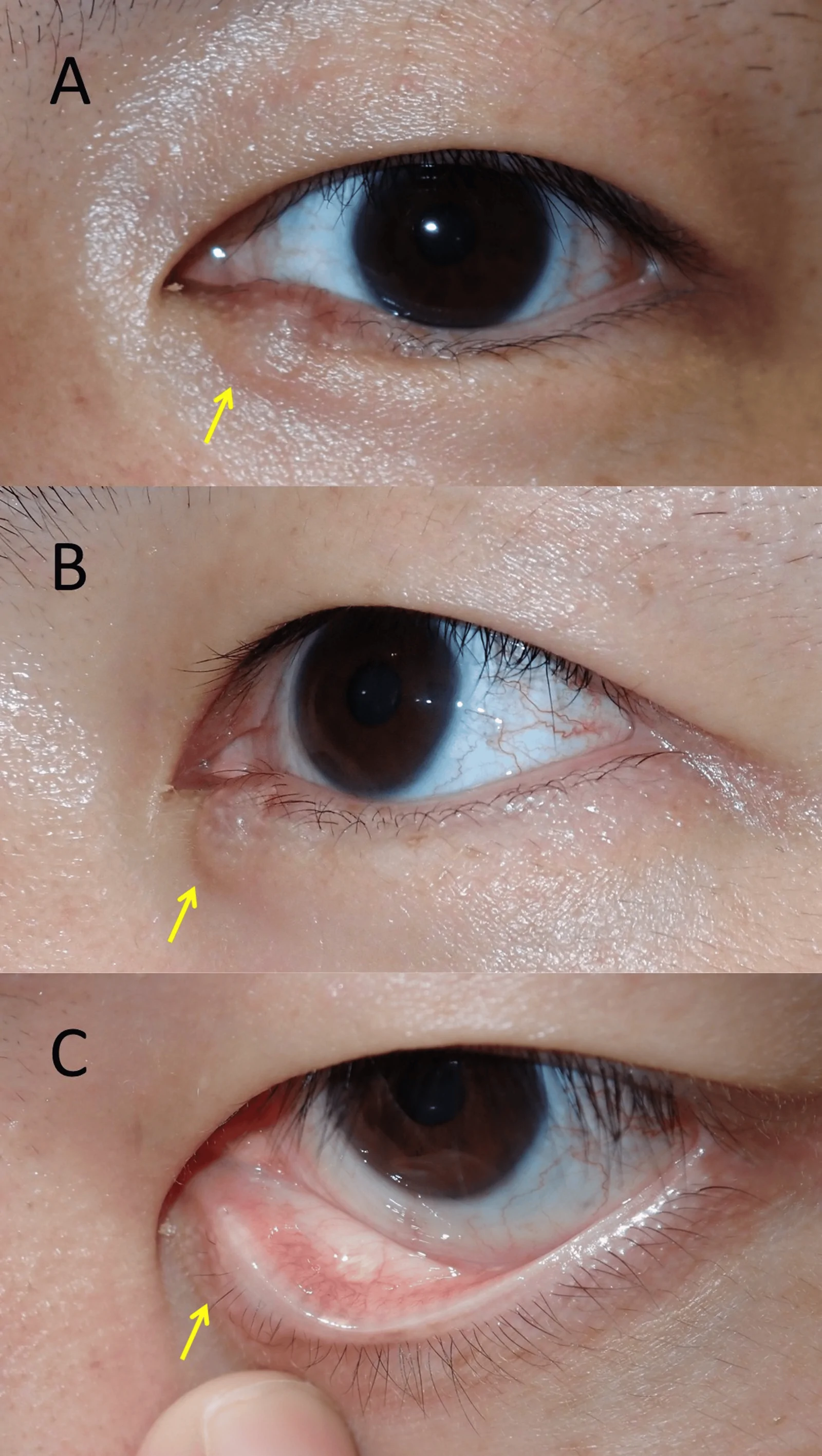
Preoperative appearance of the left lower eyelid. (A) Frontal view. (B) Oblique view. (C) Palpebral conjunctival view showing no abnormal findings. The lesion measured 7 × 7 mm on direct clinical examination; no photographic scale reference was available.

Physical examination revealed a well-circumscribed, dome-shaped, skin-colored, elastic-soft, non-tender subcutaneous mass. Direct clinical measurement demonstrated that the lesion measured approximately 7 × 7 mm (Figures 1A-1B). The superior margin of the lesion was immediately beneath the lower eyelid lash line, and the lesion was located lateral to the lower punctum. The overlying skin was intact without erythema, and the lesion was freely mobile relative to the overlying skin but showed limited mobility relative to the deeper tissue. No abnormalities of the lower eyelid margin, eyelashes, lower punctum, or palpebral conjunctiva were observed, and the patient denied epiphora (Figure 1C). Based on these findings, a benign cystic lesion of the eyelid was suspected, and excisional biopsy under local anesthesia was planned.

**Figure 2:**
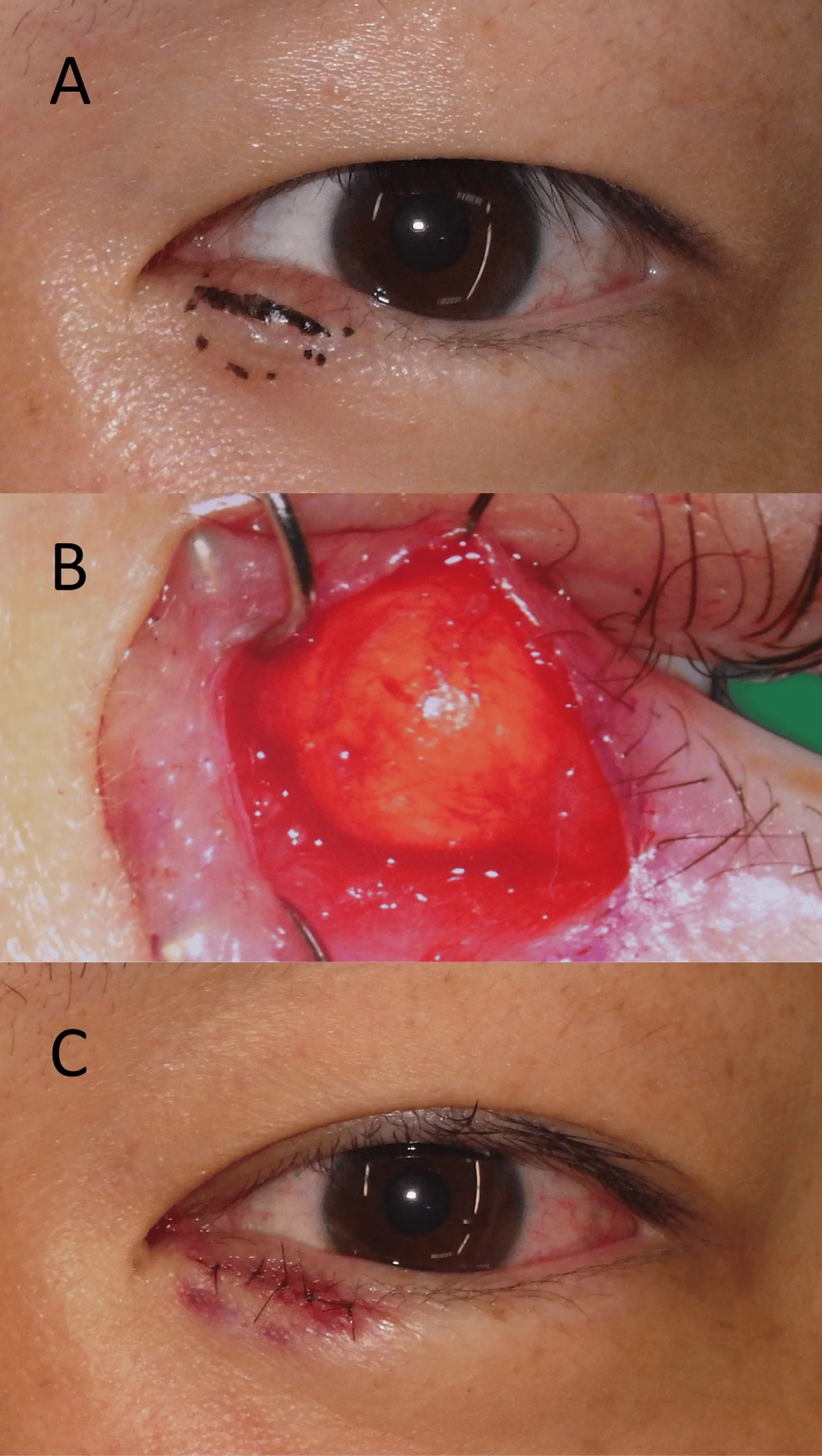
Intraoperative findings. (A) Skin incision design. (B) Intraoperative view of the exposed cystic lesion during dissection. (C) Immediate postoperative appearance after wound closure.

The lesion was excised under local anesthesia. A vertical skin incision parallel to the eyelid margin was made directly over the lesion. During dissection, the lesion was found to be closely apposed to the anterior surface of the lower tarsal plate and was carefully dissected from the surrounding tissue (Figure 2B). Before rupture, the lesion appeared as a translucent, spherical, thin-walled cyst, and direct intraoperative measurement showed that it measured approximately 7 × 7 mm. During further dissection, the thin-walled cyst was inadvertently ruptured, releasing a small amount of clear, colorless serous fluid. Following rupture, the entire circumference of the residual cyst wall was carefully identified and dissected from the surrounding tissue, with no grossly visible cyst wall remaining in the surgical field. The excised cyst wall was submitted in its entirety for histopathological examination. No gross pigmentation was identified.

**Figure 3:**
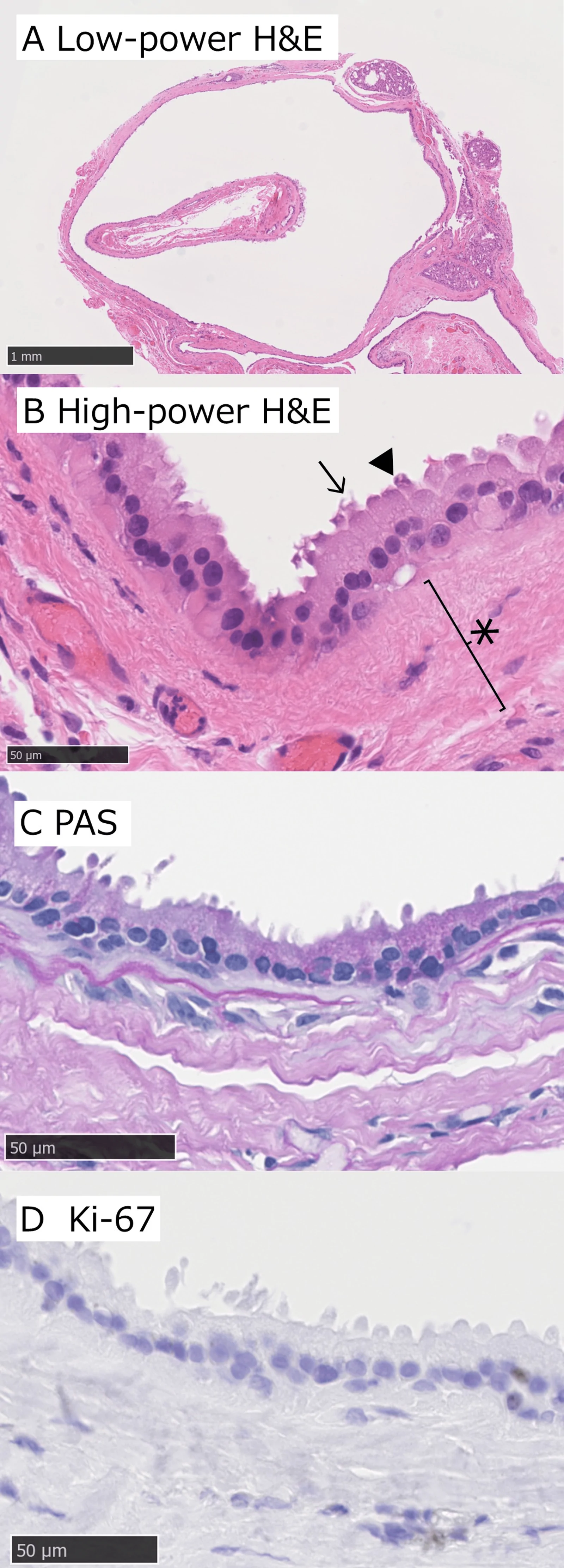
Histopathological findings. (A, B) Hematoxylin and eosin staining demonstrating a well-circumscribed unilocular cyst lined by one to two layers of cuboidal to columnar epithelial cells with papillary infoldings (arrowhead), apical decapitation secretion (arrow), and an outer myoepithelial cell layer (asterisk). (A) Original magnification ×2.5; scale bar = 1 mm. (B) Original magnification ×40; scale bar = 50 μm. (C) Periodic acid-Schiff (PAS) staining demonstrating cytoplasmic positivity in the cyst-lining epithelial cells. Original magnification ×40. Scale bar = 50 μm. (D) Immunohistochemical staining for Ki-67 demonstrating rare positively stained nuclei in the cyst-lining epithelial cells. Original magnification ×40. Scale bar = 50 μm.

Histopathological examination demonstrated a well-circumscribed unilocular cyst lined by one to two layers of cuboidal to columnar epithelial cells (Figures 3A-3B). The cyst wall exhibited characteristic papillary infoldings and apical decapitation secretion. An outer layer of myoepithelial cells with short spindle-shaped nuclei was identified (Figure 3B). No cytological atypia or mitotic figures were observed. Periodic acid-Schiff (PAS) staining demonstrated cytoplasmic positivity in the cyst-lining epithelial cells; diastase digestion was not performed (Figure 3C). Immunohistochemical staining for Ki-67, performed as part of the routine pathological evaluation, demonstrated only rare positively stained nuclei in the cyst-lining epithelial cells (Figure 3D). Based on these characteristic histopathological findings, the lesion was diagnosed as an apocrine hidrocystoma.

The postoperative course was uneventful. At the one-month follow-up, no evidence of local recurrence was observed.

## Discussion

Although apocrine hidrocystoma of the eyelid is a well-recognized entity, its clinical appearance overlaps with several common benign eyelid cystic lesions, making accurate preoperative diagnosis challenging. The present case provides an educational example of its characteristic clinicopathological features and the role of histopathological examination in establishing the definitive diagnosis.

In a large clinicopathological series of 5,504 eyelid skin tumors, 84% were benign, and hidrocystomas accounted for 8% of all cases [6]. Epidermoid cysts, hybrid cysts, intratarsal keratinous cysts, conjunctival cysts, and eccrine or apocrine hidrocystomas should be considered in the differential diagnosis of eyelid cystic lesions [5,7-9]. In addition, eccrine hidrocystomas and apocrine hidrocystomas may closely resemble other benign cystic lesions, making definitive preoperative diagnosis based solely on physical examination frequently difficult [4,5]. Although bedside findings such as transillumination may provide supportive information for some translucent cystic lesions, they are not sufficiently specific to reliably establish a definitive diagnosis because considerable clinical overlap exists among benign cystic eyelid lesions. Therefore, histopathological examination remains essential for definitive diagnosis.

Histopathologically, apocrine hidrocystoma is characterized by a cyst lined by one or two layers of cuboidal to columnar epithelial cells with papillary infoldings, apical decapitation secretion, and an outer myoepithelial cell layer derived from the glands of Moll [3,5,10]. These characteristic findings were all observed in the present case. Cytological atypia and mitotic activity were absent, further supporting the diagnosis. PAS-positive granules have been described in apocrine hidrocystomas [4]. In the present case, PAS staining showed cytoplasmic positivity in the cyst-lining epithelial cells; diastase digestion was not performed. Ki-67 immunohistochemistry, also performed as part of the routine pathological evaluation, demonstrated only rare positively stained nuclei in the cyst-lining epithelial cells. Consistent with these findings, Jakobiec and Zakka proposed that many eyelid hidrocystomas previously classified as eccrine are more appropriately regarded as lesions of apocrine (Moll gland) origin based on detailed microanatomical and immunohistochemical analyses [5].

Apocrine hidrocystomas occur over a wide age range and without a marked sex predilection [2,4]. In the analysis of 167 Japanese cases by Anzai et al., most patients were adults aged 30-69 years, and the face was the most common site (102/167 cases, 61.1%), including 55 periocular lesions [2].

Differentiation from eccrine hidrocystoma may be difficult because both lesions can present as clinically similar cystic nodules. Seasonal variation has been described more frequently in eccrine hidrocystomas, which may enlarge during hot and humid weather, whereas apocrine hidrocystomas generally lack this feature [4,5]. However, seasonal variation is not sufficiently specific to establish the diagnosis and should be regarded only as a supportive clinical clue. In the present case, the patient reported no seasonal change in lesion size. Pigmented variants may also clinically mimic malignant eyelid tumors, further underscoring the importance of histopathological examination for definitive diagnosis [4].

Complete surgical excision is commonly recommended because it permits definitive histopathological diagnosis while removing the lesion [4]. In contrast, simple aspiration or drainage may be followed by recurrence [4]. In the present case, the postoperative course was uneventful, and no recurrence was observed at the one-month follow-up. This case illustrates that apocrine hidrocystoma should be considered in the differential diagnosis of painless eyelid cystic lesions and that, although clinical findings may provide supportive clues, definitive diagnosis relies on histopathological examination.

The present report is limited by the short postoperative follow-up period; therefore, the absence of recurrence at one month should be interpreted as an early postoperative observation rather than evidence of long-term cure.

## Conclusions

Apocrine hidrocystoma should be considered in the differential diagnosis of painless cystic eyelid lesions. Because its clinical appearance may overlap with that of other benign eyelid cysts, definitive diagnosis relies on characteristic histopathological findings. This case provides an educational example of the clinicopathological features of eyelid apocrine hidrocystoma and the diagnostic role of histopathological examination.
